# Diabetes control is worse in children and young people with type 1 diabetes requiring interpreter support

**DOI:** 10.3389/fcdhc.2023.1228820

**Published:** 2023-11-27

**Authors:** Jan Idkowiak, Suma Uday, Sabba Elhag, Timothy Barrett, Renuka Dias, Melanie Kershaw, Zainaba Mohamed, Vrinda Saraff, Ruth E. Krone

**Affiliations:** ^1^ Institute of Metabolism and Systems Research, College of Medical and Dental Sciences, University of Birmingham, Birmingham, United Kingdom; ^2^ Centre for Endocrinology, Diabetes and Metabolism, Birmingham Health Partners, University of Birmingham, Birmingham, United Kingdom; ^3^ Department of Endocrinology and Diabetes, Birmingham Children’s Hospital, Birmingham Women’s and Children’s NHS Foundation Trust, Birmingham, United Kingdom; ^4^ Institute of Cancer and Genomic Sciences, College of Medical and Dental Sciences, University of Birmingham, Birmingham, United Kingdom

**Keywords:** communication barriers, language, social deprivation, diabetes outcome, hemoglobin A1c protein, type 1 diabetes

## Abstract

**Introduction:**

Language barriers can pose a significant hurdle to successfully educating children and young people with type 1 diabetes (CYPD) and their families, potentially influencing their glycaemic control.

**Methods:**

Retrospective case-control study assessing HbA1c values at 0, 3, 6, 9, 12 and 18 months post-diagnosis in 41 CYPD requiring interpreter support (INT) and 100 age-, sex- and mode-of-therapy-matched CYPD not requiring interpreter support (CTR) in our multi-diverse tertiary diabetes centre. Data were captured between 2009-2016. English indices of deprivation for each cohort are reported based on the UK 2015 census data.

**Results:**

The main languages spoken were Somali (27%), Urdu (19.5%), Romanian (17%) and Arabic (12%), but also Polish, Hindi, Tigrinya, Portuguese, Bengali and sign language. Overall deprivation was worse in the INT group according to the Index of Multiple Deprivation (IMD [median]: INT 1.642; CTR 3.741; p=0.001). The median HbA1c was higher at diagnosis in the CTR group (9.95% [85.2 mmol/mol] versus 9.0% [74.9 mmol/mol], p=0.046) but was higher in the INT group subsequently: the median HbA1c at 18 months post diagnosis was 8.3% (67.2 mmol/mol; INT) versus 7.9% (62.8 mmol/mol; CTR) (p=0.014). There was no hospitalisation secondary to diabetes-related complications in either cohorts.

**Summary and conclusions:**

Glycaemic control is worse in CYPD with language barriers. These subset of patients also come from the most deprived areas which adds to the disadvantage. Health care providers should offer tailored support for CYP/families with language barriers, including provision of diabetes-specific training for interpreters, and explore additional factors contributing to poor glycaemic control. The findings of this study suggest that poor health outcomes in CYPD with language barriers is multifactorial and warrants a multi-dimensional management approach.

## Introduction

Language concordance between physicians and patients is becoming increasingly important due to increased migration across countries and continents. The need for safe and effective communication between patients and clinicians is unquestionable. Language concordance in the healthcare setting has been shown to improve healthcare outcomes (1). Whilst effective communication between the multi-professional team and patients is the key in many chronic health conditions, it is particularly important in type 1 diabetes where the focus is delivering self-management skills to prevent acute complications from hypoglycaemia and chronic complications from hyperglycaemia alike. Language barriers have been identified to delay access to health care providers amongst immigrants, jeopardising equity of health care access (2). Children and families with language discordance are at a higher risk of harm in the hospital setting as they are less likely to speak up if treatment decisions are questionable (3). In adults with type 2 diabetes, language discordance has been identified as an independent predictor for poor glycaemic control in US Latinos, which was not observed when healthcare providers spoke the same language (4, 5). In the United Kingdom, the National Paediatric Diabetes Audit (NPDA) data highlights that the Children and Young People with Diabetes (CYPD) living in the least deprived areas had an average HbA1c 5.88 mmol/mol (0.5%) lower than those living in the most deprived areas (6, 7). Multiple factors have been attributed to poorer diabetes control in this group, including the reduced use of technology such as insulin pump therapy and continuous glucose monitors, which are likely to improve diabetes control (7). Similar disparities in healthcare and outcomes in CYPD have also been demonstrated in a German cohort (8). Additional factors such as barriers in language and cultural differences and deprivation are contributory. There is limited data available on diabetes control in CYPD and their families with communication barriers. Herein, we aim to explore the impact of language barriers on diabetes control in newly diagnosed CYPD with language barriers in our large tertiary centre in the UK, caring for patients from multi-diverse backgrounds.

## Methods

We conducted a single tertiary centre case-control study. Data were collected retrospectively between February 2009 and November 2016 on CYPD requiring interpreter support due to language barriers in either the CYP, the caregiver, or both (INT cohort). Outcome parameters were compared to a control group that did not require an interpreter (CTR).

CYP with a new diagnosis of type 1 diabetes mellitus were included, confirmed by the presence of type1-diabetes-specific antibodies (i.e. either/or anti-glutamic acid decarboxylase antibodies; anti-islet cell antibodies; anti-islet-tyrosine-phosphatase 2 antibodies). We have excluded CYP from our analysis with a diagnosis of non-type 1 diabetes, a concomitant diagnosis of hypothyroidism or coeliac disease or who received systemic glucocorticoid therapy.

All CYP with newly diagnosed type 1 diabetes were started on multiple daily injection (MDI) therapy with subcutaneous insulin injections of fast-acting (Novorapid^®^/Humalog^®^) and long-acting (Lantus^®^) insulin. All CYPD/families received comprehensive structured education, including carbohydrate counting, which was delivered by specialised paediatric diabetes nurses, dietitians and paediatric diabetes consultants. The learning materials (i.e. diabetes workbook) were provided in English language. CYP and families from the INT group were supported by a qualified language interpreter, who was present at all education sessions and subsequent 3-monthly outpatient MDT appointments.

Data were collected from individual patient case notes and existing dedicated diabetes databases. These included: age at diagnosis, gender, main language spoken in the household, ethnicity, post-code, mode of therapy and HbA1c from diagnosis until 18 months post diagnosis. The INT cohort was compared to n=100 CYPD who did not require interpreter support (CTR) and were matched for age, gender and mode of insulin therapy. HbA1c values obtained at routine outpatient clinic visits were collected from both cohorts at diagnosis and 3-month intervals until 18 months after diagnosis. HbA1c levels were determined by a turbidimetric inhibition immunoassay for haemolysed blood on the Roche Cobas c501 platform (Roche, Welwyn Garden, UK).

English Indices of Multiple Deprivation (IMD) derived from the 2015 census data published by the UK Ministry of Housing, Communities and Local Government (9). The IMD is an official measure of relative deprivation in smaller neighbourhoods, comprising a ranking of one (most deprived) to 32,844 (least deprived) small areas in England. The ranks are further divided into into five equal quintiles, where 1 is the most deprived and 5 the least deprived. The individual IMD ranking was ascertained based on the individual residential postcode in CYPD for both cohorts and grouped in quintiles.

Mann-Whitney U test for non-normally distributed data was performed to assess statistical differences between the two cohorts. A p-value below 0.05 was deemed statistically significant. The software GraphPad Prism^®^ was employed to perform statistical analysis and graphical illustration of the data.

Institutional review board approval for retrospective data review was obtained from Birmingham Women’s and Children’s (BWC) NHS Foundation Trust (reference: CARMS-00935).

## Results

### Baseline demographics and language spoken

The cohort consists of n=41 CYPD requiring interpreter support (INT) and n=100 CYPD not requiring interpreter support (CTR) (**Table 1**). There was an equal distribution of gender and there was no statistical age-difference between the cohorts. All CYPD were started on MDI therapy and 29.7% (INT)/29.0% (CTR) transitioned to continuous subcutaneous insulin infusion (CSII) therapy within the observational period (18 months post diagnosis) (**Table 1**). There were no hospitalisations due to diabetes-related complications (i.e. hypoglycaemia or DKA) in either groups and none were diagnosed with coeliac disease or hypothyroidism in the study period.

**Table 1:** Baseline demographics and Index of multiple deprivation (IMD) quintiles of the study cohorts.

	Group requiring interpreter (INT)	Group not requiring interpreter (CTR)
**Number**	41	100
**Male : Female**	21:20	50:50
Mean age at diagnosis, years* (range)	7.65 (0.7-15.4)	7.29 (0.7-15.3)
% CSII within study period*	29.7%	29.0%
Ethnic Background		
- White British	–	45 (45%)
- Other White	11 (27%)	8 (8%)
- Asian	12 (29%)	30 (30%)
- Black	13 (32%)	10 (6%)
- Any other mixed	–	5 (5%)
- Other	5 (12%)	2 (2%)
Index of Multiple Deprivation quintiles**		
- 1^st^	35 (87.5%)	61 (61%)
- 2^nd^	4 (10%)	18 (18%)
- 3^rd^	1 (2.5%)	13 (13%)
- 4^th^	–	4 (4%)
- 5^th^	–	4 (4%)

* There was no statistically significant difference between the age at diagnosis between the two study groups. ** IMD quintiles were calculated based on the 2015 census data (5).

In the INT group, the majority of CYPD were of Black, Asian and White ethnic origin (**Table 1**). The majority of the CTR group were of White, followed by Asian, and Black ethnic origin (**Table 1**).

The vast majority of CYPD in the INT cohort (87.5%) were in the lowest, most deprived IMD quintile, compared to 61% of the CTR cohort. The INT cohort were within the three lower IMD quintiles; 8% of the CTR cohort was in the two top IMD quintiles (**Table 1**).

**Figure 1:**
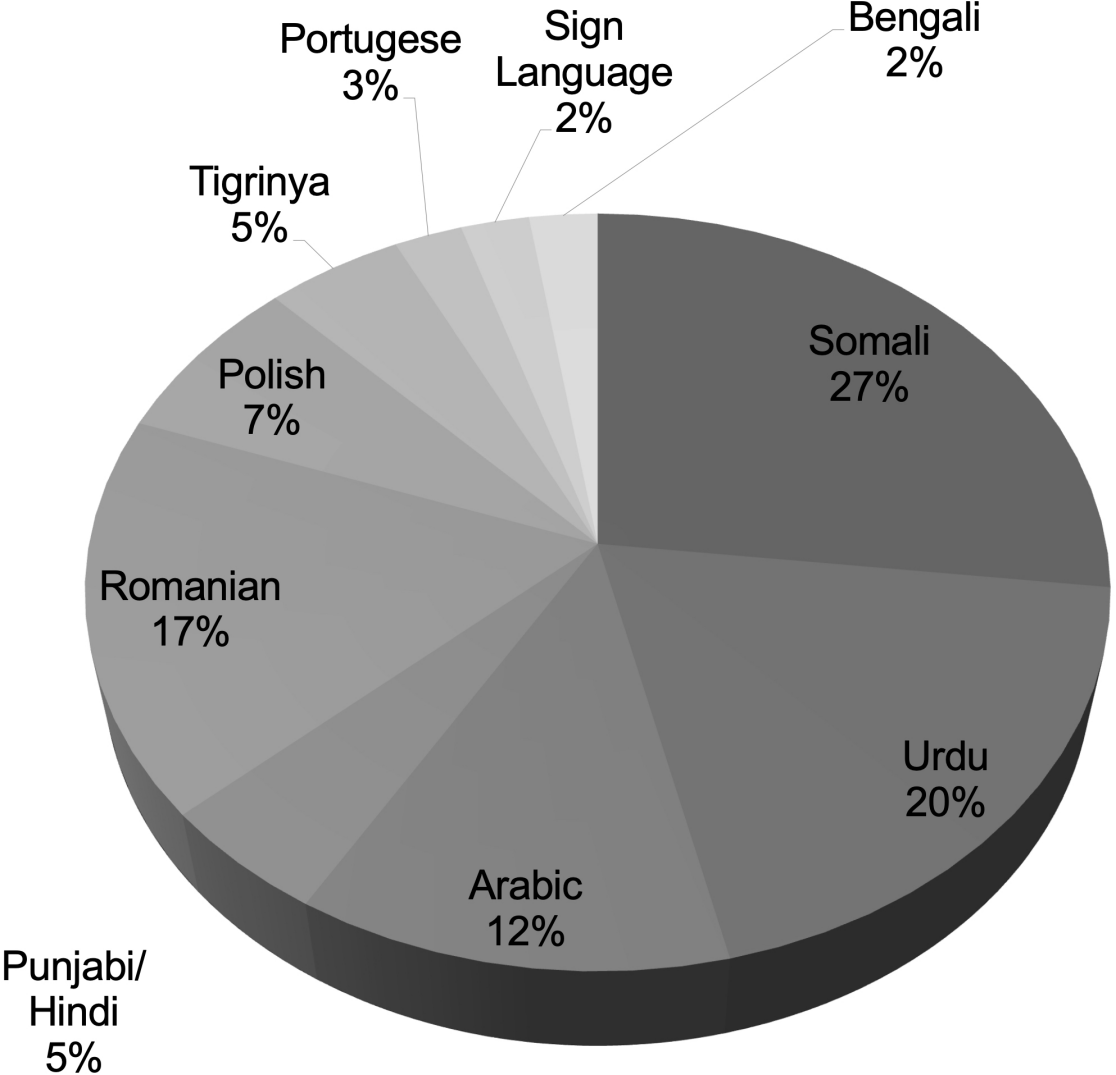
Main language spoken by the CYP or the parents/carers in our cohort of CYP with type 1 diabetes requiring interpreter support.

There were, in total, ten different languages spoken by CYP/families in the INT cohort, mainly Somali, Urdu, Romanian and Arabic (**Figure 1**).

### Glycaemic control according to HbA1c

**Figure 2:**
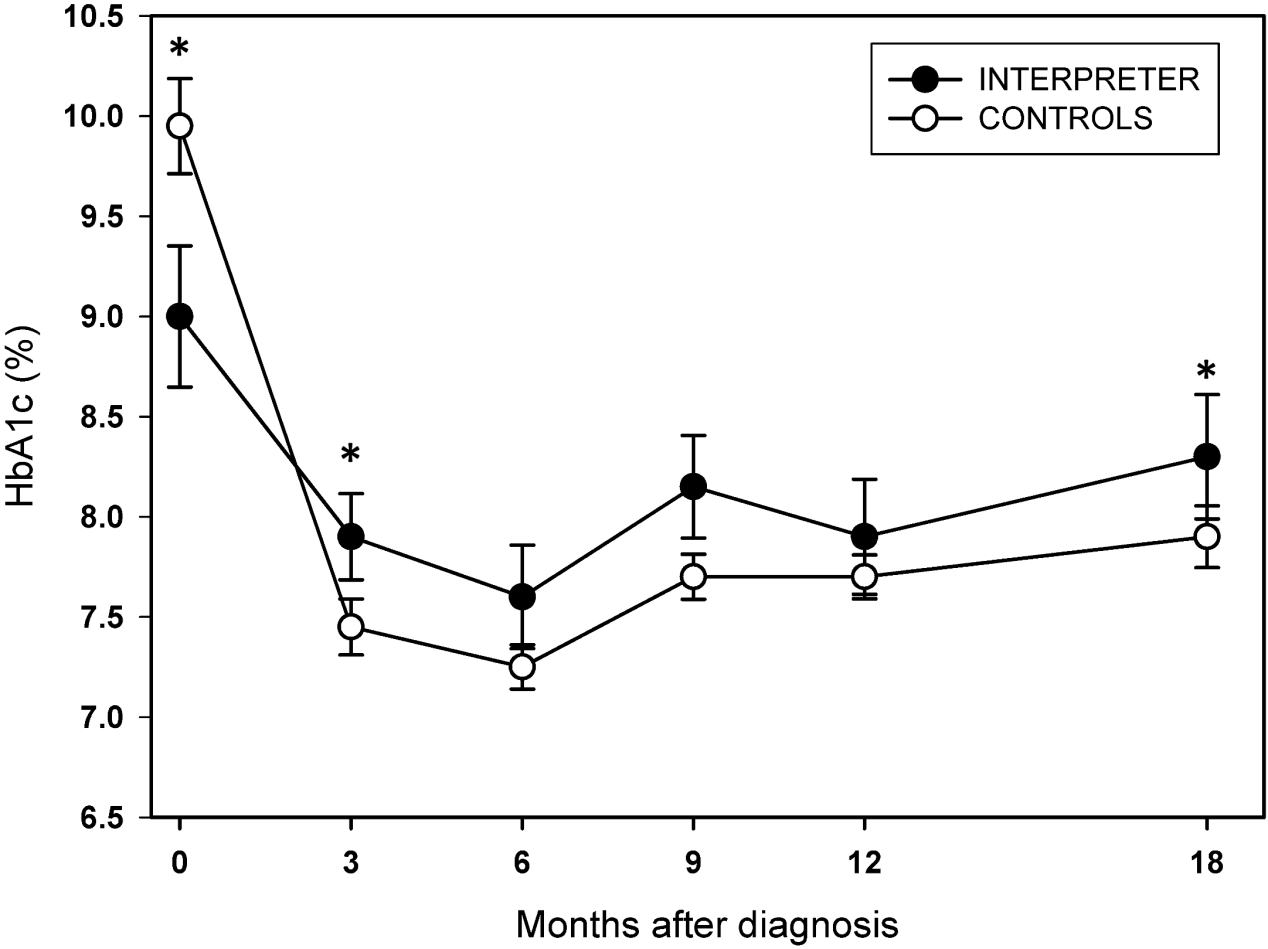
Median HbA1c (% DCCT) at diagnosis (0) and at 3, 6, 9, 12 and 18 months after diagnosis in CYPD requiring interpreter support (INT, closed circles) compared to CYPD who do not need interpreter support (CTR, open circles). Whiskers represent standard deviations for each time point. Asterisks indicate statistical significance between the two cohorts (p<0.05). To convert HbA1c from % (DCCT) to mmol/mol (IFCC): HbA1C (mmol/mol) = (A1C [%] - 2.15) x 10.929.

At diagnosis, the median HbA1c was higher in the CTR group, which was statistically significant (CTR: 9.95% [85.2 mmol/mol] versus INT: 9.0% [74.9 mmol/mol], p=0.046) (**Figure 2**). Post diagnosis, the median HbA1c was higher in the INT group, which was statistical significant at 3 months (INT: 7.9% [62.8 mmol/mol] ± 1.3; CTR 7.4% [57.4 mmol/mol] ± 1.3; p=0.036) and at 18 months (INT: 8.3% [67.2 mmol/mol] ± 1.6; CTR 7.9% [62.8 mmol/mol] ± 1.2; p=0.014) (**Figure 2**).

## Discussion

CYPD and their families requiring interpreters were likely to reside in the most deprived areas and had worse diabetes control, as assessed by HbA1c, at 18 months from diagnosis when compared to CYPD who did not require an interpreter. The gap in HbA1c in our cohort was not attributed to uptake in technology such as insulin pump therapy. Addressing linguistic barriers by utilising appropriately trained interpreters did not eliminate the gap completely.

The UK has a culturally diverse population. Our region, the West Midlands, has a higher proportion of non-white population when compared to other regions across the country. The most recent National Paediatric Diabetes Audit recorded that nearly 60% of the population in our unit were of non-white background, which is much higher than the national average, where nearly 80% are of white ethnic background (6). Additionally, 62% of our cohort are from the most deprived IMD quintile (6). Given the high proportion of ethnic minority population in our cohort, staff have a good understanding of various cultures; thus, education and advice are provided in a culturally sensitive manner, and professional interpreters are promptly engaged for every clinical encounter. However, from our experience, patients with language barriers are less likely to access our telephone advice services readily. It is recognised that any intervention tailored to ethnic minority groups must integrate aspects of culture, language, religion and health literacy skills to produce a positive impact on a range of outcomes (10). Studies which have employed these interventions systematically have also failed to demonstrate an impact on glycaemic control (11). Despite the significant technological advances in diabetes care and the overall downward trend in HbA1c, the gap in glycaemic control between those of white and non-white background remains (6, 7). Whilst there is an agreement that cultural differences in care and glycaemic control in CYPD exist and more needs to be done to address these, the most effective approach is unknown (11). The provision of learning material in different languages and diabetes-specific interpreter training to reduce technical barriers in diabetes education is a logical starting point, which we are aiming to address, but requires a significant amount of resources given the variety of languages spoken in our area.

Families requiring an interpreter were also from the most deprived quintiles, and deprivation has been directly linked to adverse diabetes outcomes (7, 8). The deprivation indices, which consider multiple dimensions, not only represent the number of deprived people living in that area but also refer to the negative consequences of the lack of facilities in that area (12). It is therefore important to understand the challenges of access to healthcare facilities in areas with a multi-ethnic population background (13) rather than studying glycaemic outcomes in isolation. Addressing the gap in care requires a multi-dimensional approach (14). Due to the relatively small number of CYPD in our cohort, the retrospective data collection and the complexity of social deprivation reflected in our region, we were not able to perform a meaningful multi-variate analysis to dissect the impact of language barriers, social deprivation indices or other factors, such as diet or socio-economic factors, on diabetes control independently. Larger, multi-centre studies or real-world data powered by registries should address such associations in future research.

To our knowledge, this is the first study exploring the impact of language discordance on diabetes control in children with type 1 diabetes. However, the subject was studied in a large cohort (n=250) of US Latinos with type 2 diabetes, and language concordance was independently associated with higher self-reported interpersonal care (5). A similar study observed significant improvement in glycemic control in Limited-English-Proficient US Latinos with type 2 diabetes once they have switched to a language-concordant health care professional (4). The facilitation of language-concordant care therefore seems an obvious strategy for diabetes management. Indeed, we propose that healthcare providers develop strategies to provide such support, which should be tailored. Equally, patients and their families should be supported to acquire language skills for ongoing diabetes education.

Communication barriers affect equitable access to health care globally and have been identified as a global public health issue (15). This highlights the need for a sustained medical and political effort toward the effective integration and support of CYPs from disadvantaged backgrounds. The new national NHS England initiative of Core20PLUS5 is an approach to support the reduction of health inequalities at both national and system level (16). The approach defines a target population cohort (‘Core20’, 20% of the most deprived population and ‘PLUS5’, range of deprived population groups including ethnic minority) and identifies ‘5’ focus clinical areas requiring accelerated improvement, of which diabetes in children and young people is a major focus (16).

In conclusion, children and young people with language barriers had worse diabetes control and resided in the deprived regions. Improving patient outcomes in ethnic minority groups requires a comprehensive approach to address cultural and linguistic barriers extending beyond language-concordant care.

## Data availability statement

The raw data supporting the conclusions of this article will be made available by the authors, without undue reservation.

## Ethics statement

The studies involving humans were approved by Birmingham Women’s and Children’s (BWC) NHS Foundation Trust (reference: CARMS-00935). The studies were conducted in accordance with the local legislation and institutional requirements. Written informed consent for participation in this study was provided by the participants’ legal guardians/next of kin.

## Author contributions

RK and JI conceptualised the study. JI, SU, SE and RK analysed the data. JI, SU and RK drafted and finalised the manuscript. All authors contributed to the article and approved the submitted version.
